# Traumatic Spinal Epidural Hematoma With Significant Neurologic Findings: A Case Report

**DOI:** 10.7759/cureus.38869

**Published:** 2023-05-11

**Authors:** Maryam K Alrazooqi, Ena Skikic, Shaikh S Iqbal, Lara Sulaiman, Omar Q Muhammed Noori

**Affiliations:** 1 Emergency Medicine, Rashid Hospital Trauma Centre/Dubai Academic Health Corporation, Dubai, ARE; 2 Radiology, Rashid Hospital Trauma Centre/Dubai Academic Health Corporation, Dubai, ARE

**Keywords:** spinal epidural hematoma (seh), traumatic spinal epidural hematoma and rheumatoid arthritis, rheumatoid arthritis, tseh, traumatic spinal epidural hematoma

## Abstract

Traumatic spinal epidural hematoma (TSEH) is a rare neurosurgical emergency. Our case report centers around a young 34-year-old female brought into our emergency department after a front and rear motor vehicle collision. Clinical deterioration and imaging studies revealed a large spinal epidural hematoma extending from levels C5 to T2. The patient was subsequently transferred to a different hospital for further management. This case involved a multidisciplinary approach by the combined effort of emergency medicine physicians, neurosurgeons, orthopedic trauma surgeons, general surgeons, radiologists, intensive care specialists, anesthesiologists, paramedics and nurses.

## Introduction

Four possible types of spinal hematomas exist: epidural, subdural, subarachnoid and intramedullary hematomas. A spinal epidural hematoma (SEH) is defined as a collection of blood between the spinal dura mater and the bony surface of vertebrae. It can be classified etiologically as being either iatrogenic, spontaneous, or traumatic. It has a significant potential in causing disability or death if not diagnosed and treated early. The lesion was first described early in the 1800s, and its reported incidence has drastically increased with the availability of modern imaging modalities, nevertheless, it remains rare to this day [1]. It is estimated to occur in only 1 per 1,000,000 people annually [2]. Moreover, a spinal epidural hematoma is associated with a staggering mortality rate that can reach as high as 50% [3]. We present a case of a spinal epidural hematoma that occurred following trauma in an adult patient.

## Case presentation

A 34-year-old woman with a known case of hypothyroidism, episodic hypocalcemia, iron deficiency anemia, and rheumatoid arthritis was brought into our emergency department by ambulance 40 minutes after sustaining injuries in a three-vehicle collision. She was the driver of the middle car, sustaining both front and rear impact while restrained. On arrival, the patient had abdominal, neck and upper back pain with weakness and numbness of all four limbs, as well as urinary incontinence. She had retrograde amnesia but was seen walking at the scene of the accident. On arrival, she was drowsy with a Glasgow Coma Scale (GCS) of 14/15. She was immobilized on a backboard with a cervical collar, head blocks, and straps.

The primary survey revealed a patent airway, cervical spine immobilized, and intact breathing with good air entry bilaterally. There were no signs of chest trauma. Blood pressure was 126/90 mmHg and heart rate was 80 bpm. Extended Focused Assessment with Sonography in Trauma (eFAST) scan was negative. GCS was 14/15 (E4V4M6), and the pupils were 3mm, equal and reactive bilaterally. The patient was slightly drowsy but oriented and answering questions. Upon exposure, no obvious deformity or external bleeding was present and log rolling revealed a tender cervical and thoracic spine.

The secondary survey revealed weakness of all four limbs (power was 2/5 on the Medical Research Council (MRC) muscle power scale), with plantar downgoing. Objective assessment of sensation showed hypoesthesia in the distal parts of all four limbs. Urinary incontinence was noted. There were no additional findings in the head, neck, chest, abdomen, or extremities.

Provisional diagnoses based on our clinical findings were set as polytrauma with a possible head or spinal injury. A computed tomography polytrauma scan (CT brain, face, c-spine, thorax, abdomen, pelvis, and dorsal spine) and labs were ordered.

A preliminary verbal report of the CT polytrauma scan did not reveal any obvious fractures or injuries of internal organs. However, when notified of the neurologic findings, the radiologist had a more detailed assessment of the images, including the soft tissue windows of the neck CT.

Reassessment of the patient at 45 minutes after arrival (approximately 1.5h after the accident) revealed a loss of pinprick sensation below the shoulder level with flaccid paralysis of all four limbs (power 0/5 on MRC muscle power scale). Approximately 10 minutes later, the patient’s GCS dropped to E1V2 (further motor response was impossible to assess due to the only remaining motor function being eye and facial movement) prompting immediate Anesthesia team involvement in order to perform rapid sequence intubation, as part of the polytrauma management. Further deterioration occurred in succession, with the patient becoming hypotensive at 80/60 mmHg and bradycardic at 35 bpm. After her heart rate was optimized with atropine, she was started on a norepinephrine infusion and intravenous (IV) fluids prior to sedation and intubation. During this sequence of events, we received a critical call from the radiology department, notifying us that the soft tissue CT window demonstrated a prevertebral hematoma at the cervicothoracic level.

**Figure 1:**
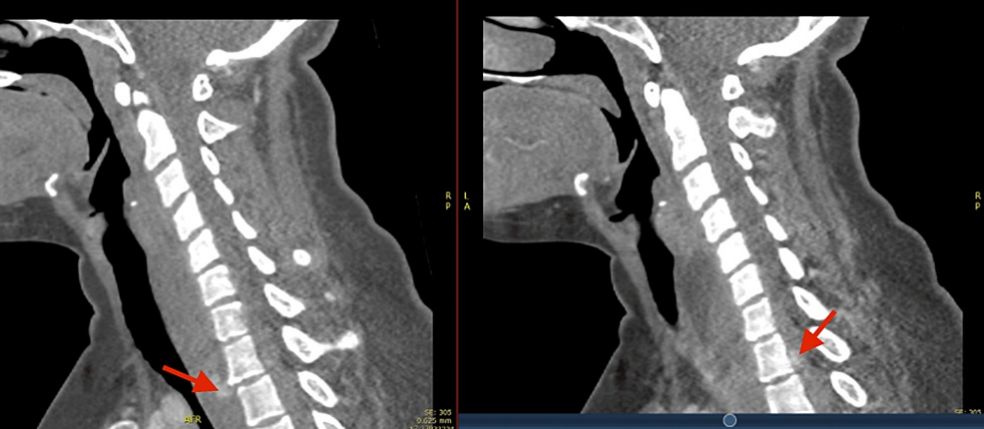
Sagittal soft tissue CT windows reveal a large prevertebral and intraspinal haematoma from level C5-T2, with specks of contrast extravasation (Red arrow).

**Figure 2:**
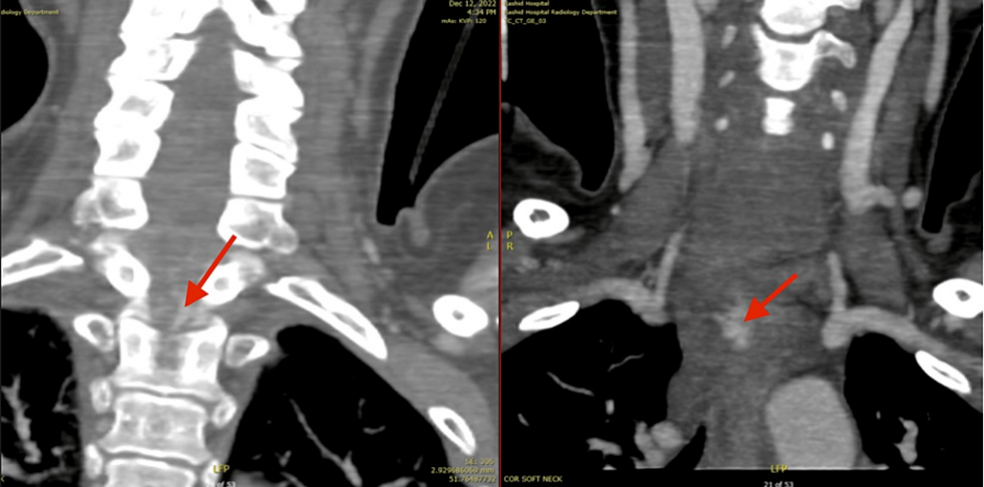
Coronal soft tissue CT windows reveal a large prevertebral and intraspinal haematoma from level C5-T2, with specks of contrast extravasation (Red arrow).

**Figure 3:**
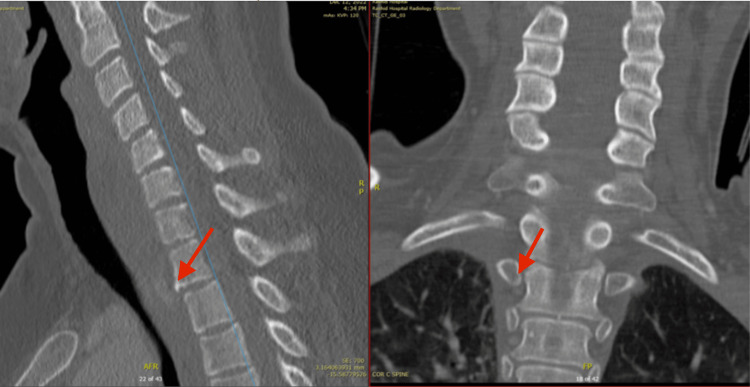
Sagittal and coronal bone reconstructed CT images of the cervical spine with chip fracture of T1 anterio-inferior endplate and rib fractures.

The CT polytrauma findings included (See Figures 1-3): A moderate to severe whiplash cervical spine injury with prevertebral hematoma from C5 to T2 vertebral levels. There were small specks of active contrast extravasation in the prevertebral region (C7-T1 levels) and posterior to the T1 vertebral body in the spinal canal with highly suspicious intraspinal hematoma. Fractures of the first and second ribs at the costovertebral junction of T1 and T2 were noted, as well as a small chip fracture of the anteroinferior endplate of T1 vertebral body. Urgent magnetic resonance imaging (MRI) of the cervical and thoracic spine was ordered.

Laboratory findings at this time were clinically unremarkable (see Table 1) with the exception of a hemoglobin level of 10 g/dL and a calcium level of 6.9 mg/dL (patient was given calcium gluconate 2 grams IV). Her coagulation profile was well within normal limits.

**Table 1:** Laboratory values at presentation with respective reference ranges (V = venous blood) WBC: White blood cells; RBC: Red blood cells; Hgb: Hemoglobin; PT: Prothrombin time; aPTT: Activated partial thromboplastin time; INR: International normalized ratio; CPK: Creatine phosphokinase; eGFR: estimated glomerular filtration rate; K: Potassium; Na: Sodium; Ca: Calcium; Cl: Chloride; pH: potential of hydrogen; pCO2: partial pressure of carbon dioxide; pO2: partial pressure of oxygen; HCO3:  Bicarbonate; ALT: Alanine transaminase; T4: Thyroxine; TSH: Thyroid stimulating hormone.

Laboratory parameter	Value	Reference range
WBC	4.3 x 10³/µL	3.6-11.0 x 10³/µL
RBC	3.19 x 10⁶/µL	3.6-11.0 x 10⁶/µL
Hgb	10.0 g/dL	12.0-15.0 g/dL
Hematocrit	31.0%	36-46%
Platelets	204 x 10³/µL	150-410 x 10⁶/µL
PT	13.5 s	11-14 seconds
aPTT	36.6 s	28-41 seconds
INR	1.02	0.8-1.1
CPK	144 U/L	<167 U/L
Troponin	<3.0 ng/L	<14 ng/L
Lactic acid	1.3 mmol/L	0.5-2.2 mmol/L
Glucose (random)	101 mg/dL	65-140 mg/dL
Urea	34 mg/dL	12-40 mg/dL
Creatinine	0.7 mg/dL	0.5-0.9 mg/dL
eGFR	116.3 mL/min	>60 mL/min
K	4.6 mmol/L	3.4-5.0 mmol/L
Na	136 mmol/L	134-143 mmol/L
Ca	6.9 mg/dL	8.9-10.2 mg/dL
Cl	105 mmol/L	97-108 mmol/L
pH (V)	7.403	7.35-7.45
pCO2 (V)	36.2 mmHg	40-50 mmHg
pO2 (V)	55.4 mmHg	30-55 mmHg
HCO3 (V)	22.6 mmol/L	21-28 mmol/L
Alkaline Phosphatase	68 U/L	35-104 U/L
ALT	43 U/L	0-31 U/L
Total Protein	6.9 g/dL	6.6-8.7 g/dL
T4	11.3 pmol/L	11.0-22.0 pmol/L
TSH	1.250 uIU/mL	0.3-4.2 uIU/mL

Diagnosis and management

Traumatic spinal epidural hematoma (TSEH) was initially clinically suspected and radiologically confirmed after the patient was stabilized.

**Figure 4:**
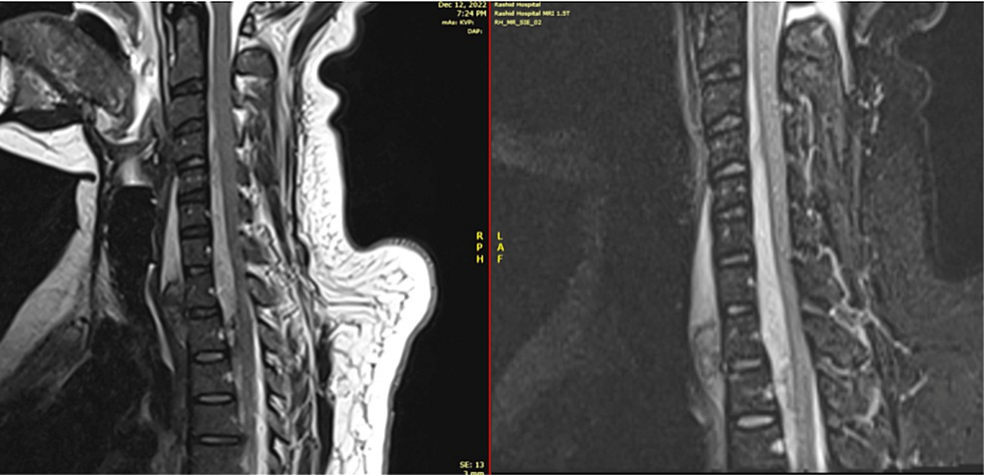
MRI sagittal T2 and STIR images reveal large prevertebral and intraspinal epidural hematoma (C5-T2) with spinal cord compression. STIR: Short Tau Inversion Recovery

**Figure 5:**
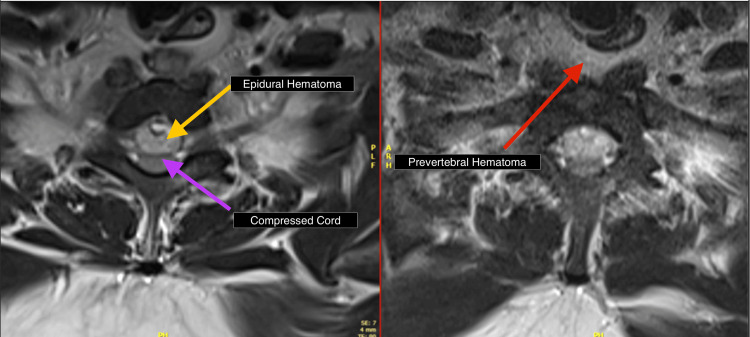
MRI Axial T2WI images reveal large prevertebral and intraspinal epidural hematoma with spinal cord compression.

**Figure 6:**
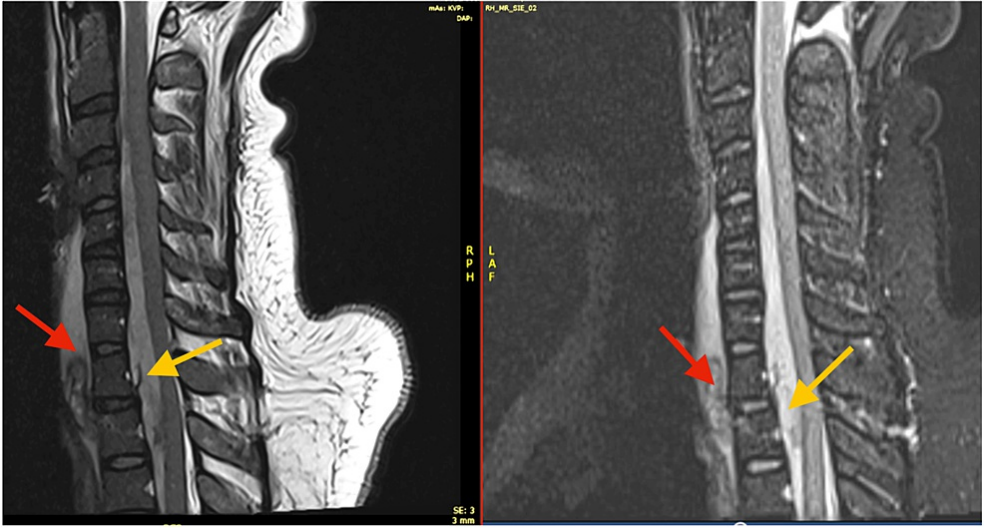
MRI SAG T2 and STIR images reveal large prevertebral (red arrow) and intraspinal epidural (yellow arrow) hematoma (C5-T2) with spinal cord compression. STIR: Short Tau Inversion Recovery; SAG: Sagittal

**Figure 7:**
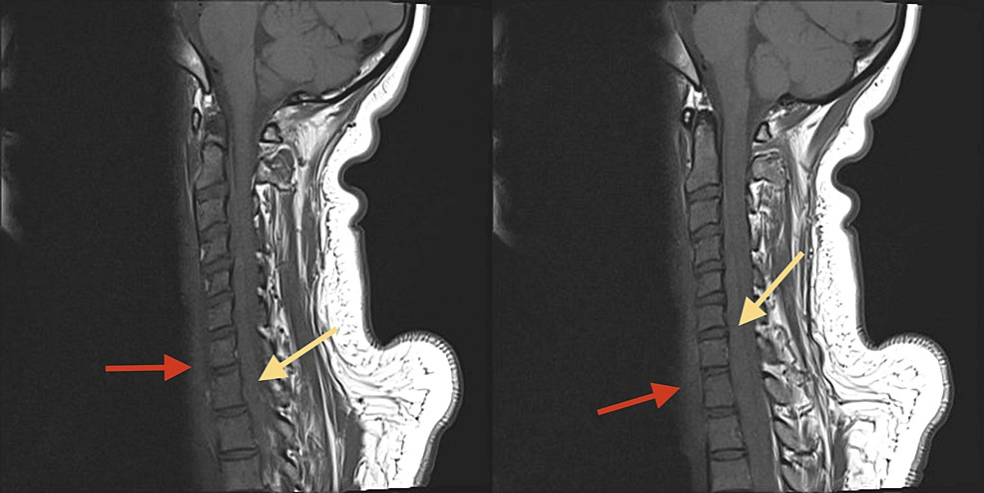
MRI SAG T1 WI reveals slightly hyperintense prevertebral (red arrow) and intraspinal haematoma (yellow arrow). SAG: Sagittal

MRI of the cervical and thoracic spine ultimately revealed (See Figures 4-7): A large epidural hematoma (EDH) extended from level C5 to T2, causing moderate compression of the cervical spinal cord. No cord edema, cord contusion, or myelopathic changes were present. A moderate prevertebral hematoma between C5 and T2 with areas of active bleed was noted. Small chip fractures of T1 and T2 vertebral bodies were observed with minimal bone marrow edema, associated with anterior and posterior longitudinal ligament tears at the same level.

The departments of Neurosurgery and Orthopedic Trauma Surgery were notified and they, in conjunction with the Surgical ICU, discussed the possible approaches in the management of such a case. According to our neurosurgical and spinal surgical teams, evacuation of this SEH would require multi-level anterior discectomies and vertebrectomies along the length of the collection, which measured 11cm from level C5 to T2. Additionally, the imaging findings revealed an active bleed without a known source, further risking re-collection of the hematoma and possible hastening of the patient’s deterioration. They deemed the surgery too risky in this case and explained to the family that the risks outweigh the benefits of attempting the procedure. Conservative management at our facility was offered, namely steroid administration and supportive management. The family was otherwise counseled regarding alternative treatment options in other medical facilities in Dubai. The family made their final decision in favor of a surgical procedure in another healthcare facility. Based on the family’s wishes, the patient was transferred to another facility, however, she unfortunately died of complications three days later. Her medical records from the other hospital were unavailable to the research team.

## Discussion

In 1682, Duverney first described a spinal hematoma in the context of a postmortem finding, while in 1869, Jackson et al. published a report of the first clinical diagnosis of the entity [4]. There are four types of spinal hematomas: epidural, subdural, subarachnoid, and intramedullary hematomas [5]. Spinal epidural hematoma (SEH) is a rare condition affecting 1 per 1,000,000 persons a year [2] with a dominant male-to-female ratio of 1.4:1 [3] and mostly affecting individuals between the ages of 42 and 52 years [3].

A spinal epidural hematoma is a collection of blood between the spinal dura mater and the bony surface of the vertebral spine. These lesions are commonly associated with factors that include but are not limited to, trauma, coagulopathies, and iatrogenic interventions [6]. Iatrogenic causes of SEH include epidural and spinal blocks, spinal manipulation, acupuncture, and discectomies. These lesions may also arise spontaneously and represent about 40% of all epidural bleeding [7]. Spontaneous SEH occurs either when there is a rise in intraspinal pressure due to a straining activity, usually in combination with a predisposing factor. These seemingly benign activities may include weight lifting, labor, prolonged valsalva maneuvers, or stretching. Some spontaneous hematoma cases that have been described are idiopathic in origin, without any clear underlying pathology. Finally, the third etiological source of spinal epidural hematomas is trauma.

Traumatic spinal epidural hematomas (TSEH) occur mostly in adults over the age of 40 years, mostly located in the cervical and proximal thoracic spine (C5-T2). It is often localized posteriorly, making anterior localizations a rare occurrence. It occurs in 0.5% to 1.7% of all spinal injuries but the incidence rises to 9% in cases of patients with concurrent rheumatic diseases such as ankylosing spondylitis, rheumatoid arthritis, and psoriatic spondylitis [7]. The link between these comorbidities is poorly understood. When associated with vertebral fractures they generally occur from high energy impact in combination with one or more predisposing factors contributing to the lesion formation.

While it is speculated that there is a greater number of SEH which are asymptomatic and simply resolving undiscovered, symptomatic spinal epidural hematomas present as sudden back or radicular pain, slowly progressing to paresis or paresthesias, and inevitably leading to para- or quadriplegia, depending on where the spinal compression occurs. This distinctive clinical course led Markham et al. to call it 'the syndrome of spinal epidural hematoma' [8].

Certain risk factors and predisposing disorders have been identified throughout articles in the literature. The most obvious ones are factors contributing to bleeding diathesis. The next ingredient to consider is narrowing or restriction of the spinal canal space, causing a quicker and more dramatic rise in the pressure exerted by hematoma formation. Longstanding or repeated increased intraabdominal and intrathecal pressure caused by pregnancy, ascites, and bulimia nervosa, might contribute to hematoma formation, as would arteriovenous malformations and states of systemic vasculitis which accentuate the fragility of blood vessels. Finally, one consistently trending contributing factor is inflammation in the form of systemic and rheumatic disorders such as rheumatoid arthritis, ankylosing spondylitis, psoriatic arthritis, and systemic lupus erythematosus, all of which are hypothesized to affect collagenous structures such as dura mater and vascular walls, through a systemic inflammatory process [9].

Our case of SEH occurred in the context of polytrauma. Our patient was a 34-year-old woman with the only significant risk factor being a history of rheumatoid arthritis (RA). Especially important to highlight is that our patient was not on anticoagulants, nor did she suffer from any coagulopathies, excluding a major risk factor of TSEH formation. The resulting hematoma was located in the cervicothoracic region, at the anterior aspect of the spinal canal, a feature rarely seen.

Moreover, of significance to the clinical timeline is the specific vascular source of epidural bleeding. Anatomically speaking, the epidural space is rich in blood supply which is sourced from the vertebral and anterior spinal arteries through arterial arcades of anastomoses, despite which, there are watershed areas vulnerable to ischemia. Onwards, venous drainage occurs via a rich, valveless, epidural venous plexus of Batson. There is a general understanding that arterial sources are more likely to be responsible for symptoms that progress rapidly, while venous lesions are much slower to expand [10]. The CT images in our case were taken during a mixed arterial and venous phase, making it difficult to determine the source of the aforementioned extravasation. Nevertheless, the rather rapid deterioration of our patient (1.5 hours after injury), versus the typically slower deterioration of venous bleeds (15-72 hours) [11], clinically signifies a more likely arterial bleed.

Magnetic resonance imaging (MRI) has emerged as the diagnostic study of choice for spinal epidural hematomas [12], as it is the ultimate imaging modality to show the exact level and size of the spinal hematoma, as well as the most critical point of spinal cord compression. A hematoma, while hyperacute, within the first 24 hours of formation appears hypointense on T1 weighted imaging, and hyperintense on T2 [13]. Varying levels of spinal cord compression might be seen, depending on the size and location of the hematoma. The position of the hematoma in relation to the spinal canal is usually found posteriorly. In fact, Groen described the location of spontaneous SEHs as posterior or posterolateral in the spinal canal in 84% of cases in the literature reviewed, while anterior or anterolateral lesions represented only 12%. Our patient’s anterior hematoma location places her within this 12% of the affected population. The mean length of hematomas described was 4.2-5.4 vertebral segments, with most cases appearing in the cervicothoracic vertebral level range from C3 to T1 [14].

## Conclusions

Despite their rarity, traumatic spinal epidural hematomas are a significant source of morbidity and mortality in affected individuals, whether in the setting of trauma, medical intervention, or spontaneous occurrence. They are a significant cause of neurologic disability if left unrecognized or untreated in a timely manner. Magnetic resonance imaging is the diagnostic modality of choice and prompt surgical intervention with decompressive discectomies is the ultimate management in cases of trauma and significant neurological impairment. Nevertheless, the authors believe that a focused approach on raising awareness and training physicians to better suspect and recognize significant spinal cord injuries in their early stages, specifically spinal epidural hematomas, would contribute to a more positive outcome for the afflicted. Proper assessment and serial reassessment of neurologic function in the emergency department setting cannot be replaced as the ultimate first step in the diagnosis. The process of recognition also includes prompting for further and more fitting imaging studies whenever clinical suspicion arises, despite a lack of obvious spinal injury on X-ray and CT scans.
